# A Randomized Double‐Blind Controlled Evaluation of the Therapeutic Benefits of an Herbal Lip Hydrant

**DOI:** 10.1111/jocd.70041

**Published:** 2025-02-27

**Authors:** Pimchanok Sutthiboonyapan, Nicharat Sriratanasak, Bhurichaya Innets, Nachon Angkanaporn, Parinnapa Suntornchot, Warunya Panyain, Thantrira Porntaveetus, Paswach Wiriyakijja, Pithi Chanvorachote

**Affiliations:** ^1^ Department of Periodontology, Faculty of Dentistry Chulalongkorn University Bangkok Thailand; ^2^ Center of Excellence in Periodontal Disease and Dental Implant Chulalongkorn University Bangkok Thailand; ^3^ Center of Excellence in Genomics and Precision Dentistry Chulalongkorn University Bangkok Thailand; ^4^ Center of Excellence in Cancer Cell and Molecular Biology, Faculty of Pharmaceutical Sciences Chulalongkorn University Bangkok Thailand; ^5^ Department of Pharmacology and Physiology, Faculty of Pharmaceutical Sciences Chulalongkorn University Bangkok Thailand; ^6^ Faculty of Dentistry Chulalongkorn University Bangkok Thailand; ^7^ Department of Physiology, Faculty of Dentistry Chulalongkorn University Bangkok Thailand; ^8^ Department of Oral Medicine, Faculty of Dentistry Chulalongkorn University Bangkok Thailand; ^9^ Avatar Biotechnologies for Oral Health and Healthy Longevity Research Unit Chulalongkorn University Bangkok Thailand

**Keywords:** aging, antioxidant, cheilitis, herbal lip hydrant, lip roughness, prevention, stemness

## Abstract

**Objective:**

Dry and chapped lips adversely affect both lip structure and function, yet there is no established gold standard for their treatment. Herbal extracts present a promising alternative due to their natural properties, though their therapeutic potential for lip care remains underexplored. This study aims to develop and clinically evaluate a novel lip hydrant formulated with polyherbal extracts, with the objective of offering an effective solution for managing dry and chapped lips.

**Methods:**

Six herbal extracts and eight mixtures, consisting of at least three herbal extracts, were evaluated for cytotoxicity using the MTT assay in HaCaT cells. The essential molecular markers were examined by western blot analysis and immunofluorescence assay. The selected mixture was formulated into lip hydrant and tested in a randomized, double‐blind, controlled clinical trial. The 66 Thai participants with dry lip concerns were randomly assigned into two groups. Each participant applied either the lip hydrant (test group) or petroleum gel (control group) once daily for 28 days. Clinical assessments were performed at baseline and on day 28 post‐application. The lip conditions, lip texture wrinkles, and hemoglobin levels were measured. Participant assessments included ratings of lip dryness, appearance, and product satisfaction.

**Results:**

The herbal extracts demonstrated potential in strengthening cell adherence, providing antioxidant effect, and inducing self‐renewal. The Mix2 shows the most promising activity, increasing adherent protein and stemness properties, and was selected as the active ingredient for the clinical trial. In the trial, both the test and control groups experienced a significant reduction in lip roughness by day 28 compared to baseline (*p* < 0.05). However, the test group exhibited a significantly greater reduction in chapped lips than the control group (*p* < 0.05). No significant differences were found between the groups in terms of lip texture, wrinkle levels, or hemoglobin levels. Notably, both groups showed significant improvements in perceived lip dryness by day 28 (*p* < 0.001).

**Conclusion:**

The study findings support the therapeutic potential of the novel polyherbal lip hydrant in improving lip hydration, reducing roughness, and alleviating chapped lips (ClinicalTrials.gov ID: NCT06475482).

## 1 Introduction

The integrity of the lip barrier is essential for protecting against environmental stressors, pathogens, and moisture loss, which are critical for maintaining hydration and preventing damage. A well‐maintained lip barrier helps avoid sensitivity and conditions like cheilitis while preserving the aesthetic and functional integrity needed for speaking and eating. The lip is composed of three distinct zones: the labial, vermillion, and oral mucosa [1]. The vermillion, which separates the surrounding skin from the oral mucosa, is highly exposed to environmental factors and changes with age [2]. With aging, lips become thinner and drier, develop deeper vertical wrinkles, and lose color [3]. Consequently, they are frequently targeted for cosmetic and pharmaceutical treatments. Common issues include dryness, chapping, and hyperpigmentation. Dryness and chapping result from a reduced water‐holding capacity of lip skin [4], while hyperpigmentation is caused by oxidative species and inflammation [5, 6]. The growing market for herbal lip care products reflects the increasing demand for natural solutions that offer antioxidants, anti‐inflammatory, and emollient properties.

The lip vermilion is comprised of a stratified squamous epithelium with underlying tissue structures [2, 7]. This epithelium includes layers such as the stratum corneum, stratum granulosum, stratum spinosum, and stratum basale. Similar to the skin epidermis, the lip vermilion undergoes continuous renewal through the activity of skin cells located in the stratum basale [8]. The renewal process is regulated by stem cells, which play a crucial role in maintaining the integrity of the epidermal barrier [9]. Transcription factors like Oct4 and Nanog are pivotal in controlling stem cell identity, influencing the renewal rate and overall barrier function. Additionally, the adhesion molecule E‐cadherin is vital for epidermal barrier function, with its loss leading to impaired cell junctions and increased apoptosis [10].

Thai native herbs, rich in bioactive compounds, have long been utilized in traditional and alternative medicine. Peppermint oil, extracted from *Mentha piperita* L., exhibits significant anti‐inflammatory effects, largely due to its primary component, menthol, which modulates cytokine profiles by reducing pro‐inflammatory and enhancing anti‐inflammatory cytokines [11]. Clinical studies have validated the efficacy of topical and transdermal peppermint oil in managing chronic pruritus without adverse effects [12, 13]. Emblica ( *Phyllanthus emblica* L.), also known as Indian gooseberry, is renowned for its high levels of phenolic antioxidants such as flavonoids, gallic acid, and vitamin C, which are effective in preventing oxidative stress‐induced skin aging [14, 15]. *Perilla frutescens* L., part of the Lamiaceae family, contains extracts rich in anthocyanins, flavonoids, and phenolics, which inhibit redox‐sensitive signaling pathways, reducing the expression of inflammatory mediators [16]. Galangal (*Alpinia galangal* L.), a pungent aromatic rhizome, has been extensively studied for its biological activities, including antitumor, antibacterial, antiulcer, antifungal, and insecticidal properties [17]. Thus, Thai herbs offer a promising alternative due to their active natural compounds and demonstrated benefits in maintaining skin moisture, elasticity, and anti‐aging properties.

Despite the significant impact of damaged lips and cheilitis on lip structure and function, there is no universally accepted treatment. While many over‐the‐counter lip care products focus on cosmetic benefits, the therapeutic potential of herbal extracts remains underexplored. This study aims to develop and clinically evaluate a novel lip hydrant featuring polyherbal extracts, with the goal of providing an effective treatment for dry and chapped lips.

## 2 Materials and Methods

### 2.1 Herbal Extracts Preparation and Product Development

In this study, six herbs—peppermint oil (PP), perilla extract (PE), emblica fruit extract (EF), guava leaf oil (GL), galanga rhizome extract (GR), and green tea leaf extract (GT)—were used. The herbs were processed according to a standardized protocol provided by the manufacturer (TCFF). Briefly, the herbs were thoroughly washed and immersed in a solvent for liquid extraction. The extracts were subsequently separated using filtration and evaporation methods. For oil extraction, the herbs underwent steam distillation under controlled conditions of temperature, time, and pressure, followed by filtration. The final extracts were packaged under controlled conditions and subjected to quality control. These extracts were then evaluated for toxicity, antioxidant, anti‐inflammatory properties, as well as cell adherence in vitro, to develop a potential formulation.

### 2.2 Ethical Consideration

The study was approved by the Human Ethics Research Committee of the Faculty of Dentistry, Chulalongkorn University (HREC‐DCU2023‐060). All participants provided written informed consent after receiving detailed information about the study. The research was conducted in accordance with the principles outlined in the Declaration of Helsinki and reported following the CONSORT 2010 guidelines. The study was registered at ClinicalTrials.gov (ID: NCT06475482).

### 2.3 Cell Culture

The human keratinocyte (HaCaT) cells are purchased from Cell Lines Service (Heidelberg, Germany). Cells are cultured in Dulbecco's Modified Eagle's Medium (DMEM, Gibco, Grand Island, NY, USA). The medium is supplemented with 10% fetal bovine serum (FBS), 2 mM L‐glutamine, and 100 units/mL of each penicillin and streptomycin (Gibco, MD, USA) at 37°C with 5% CO_2_ in a humidified incubator.

### 2.4 Molecular and Cellular Studies

Cell viability assay: MTT assay was employed to indirectly illustrate the viable cells at 24 h. Cells were seeded at density of 1 × 10^4^ cells per wells in 96‐well‐plate. Then, the cells were treated with various concentrations of each herbal for 24 h. After that, the cells were incubated with 4 mg/mL MTT (3‐(4,5‐dimethylthiazol‐2‐yl)‐2,5‐diphenyltetrazolium bromide) solution for 3 h at 37°C. Formazan crystals were solubilized using DMSO. Subsequently, the absorbance is examined by microplate reader at 540 mm.

Immunofluorescence staining: After treatment, they were fixed with 4% paraformaldehyde in PBS for 15 min, permeabilized for 5 min with 0.5% Triton‐X, and blocked with 0.1% Triton‐X in 10% FBD in PBS for 1 h at room temperature. Cells were incubated with E‐cadherin and Oct4 primary antibodies at 4°C overnight. After that, they were incubated with secondary antibody for 1 h and stained with Hoechst 33342 for 30 min at room temperature in the dark. The images were visualized under a fluorescent microscope (Olympus DP70, Melville, NY, USA) and analyzed by the ImageJ software.

Western Blot Analysis: Western blot analysis was used to detect the presence of specific proteins. Cells were lysed in lysis buffer. The concentration of protein in each sample was analyzed by BCA assay. The extracted proteins were separated by sodium dodecyl sulfate polyacrylamide gel electrophoresis (SDS‐PAGE) and transferred to polyvinylidene difluoride membrane (PVDF). Then, the membranes were blocked with 5% non‐fat milk powder. After that, they were incubated with primary antibodies against Nrf2, E‐cadherin, Nanog, and Sox2 overnight. The membranes were washed with Tris‐buffered saline/Tween 20 and incubated with secondary antibodies. Finally, the protein bands were utilized to detect immunoreactive proteins which were further exposed to X‐ray film. The protein band intensity was examined using ImageJ software.

### 2.5 Lip Formulation

The formulation of lip hydrant base was provided as follows (Table 1):

**TABLE 1:** The ingredients in lip hydrant base were described.

Ingredients	INCI name	% w/w	Function
DI water	Aqua	qs. 100	Diluent
Xanthan gum	XANTHAN GUM	0.30	Thickening agent
Glycerin 99.5% USP	Glycerin	5.00	Humectant
Ceto stearyl alcohol 50:50	Cetearyl alcohol	7.00	Emulsifier
Herbal mixture^a^	—	5.00	Active ingredient
Spectrastat BHL	Caprylhydroxamic acid (and) 1,2‐Hexanediol (and) Butylene glycol	2.00	Preservative‐free

*Note:* All lip hydrant conditions with different mixtures were used in the same base ointment.

^a^ The herbal mixtures were varied according to the composition of each mixture in Figure 6.

### 2.6 Participants and Study Design

Sixty‐six Thai adults participated in this randomized, double‐blind, controlled clinical trial during January 1–March 27, 2024. The sample size was determined from the mean dryness score, with an error margin of 1.5, a type I error, and a power of 0.8, yielding a required sample size of 28 participants. To account for an estimated 10% dropout rate, the final sample size was adjusted to include at least 31 participants.

The inclusion criteria were participants aged 18–40 years with self‐reported dry lips who agreed to comply with the study protocol, refrain from using any other lip products during the study, and provide written informed consent. Exclusion criteria included visible skin conditions that could interfere with assessments, a history of sensitivities to cosmetic products or moisturizers, known or suspected intolerance to herbal products, recent or ongoing medication use, and conditions other than cheilitis simplex.

**FIGURE 1:**
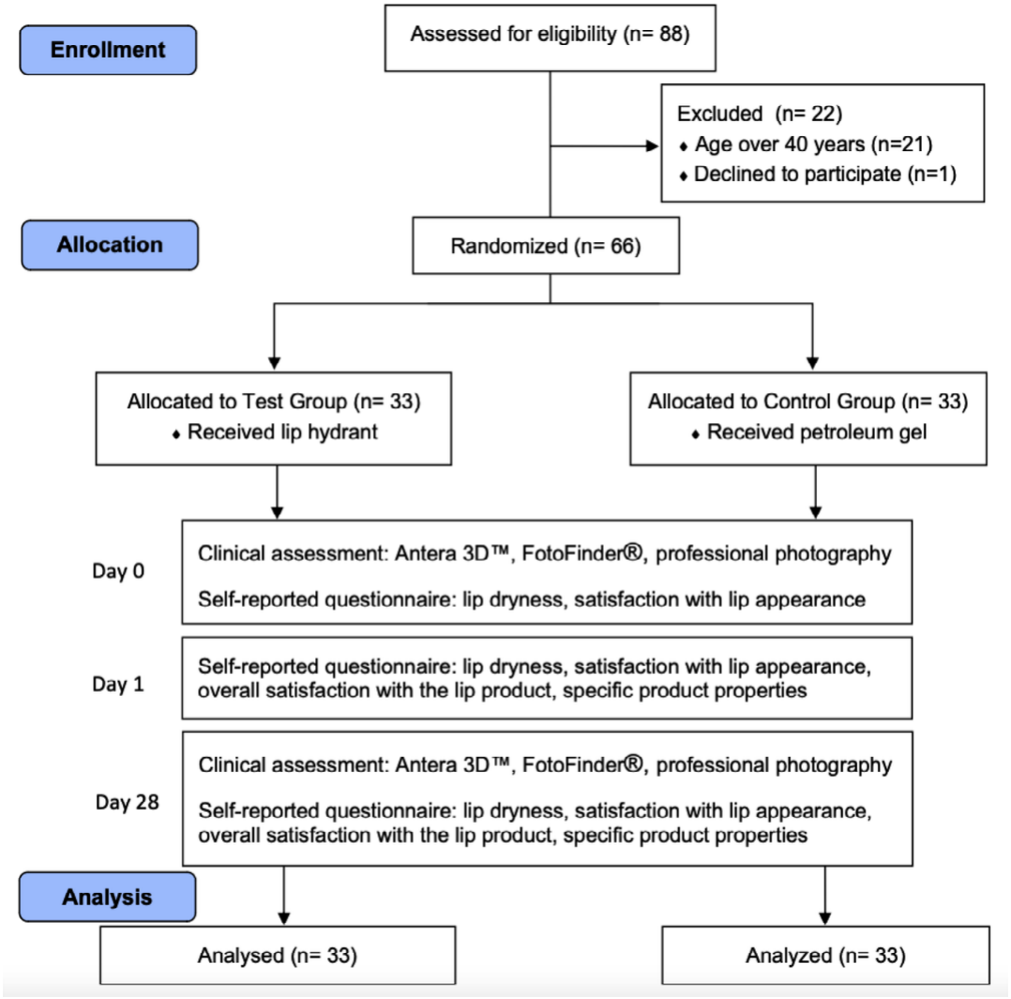
CONSORT study flow diagram.

Participants were randomly assigned to one of two groups (*n* = 33 per group) using block randomization by an investigator (P.S.) (Figure 1). Sequentially numbered product containers (01‐66) were prepared, and each participant received the corresponding container according to their recruitment sequence. The test group received the lip hydrant containing the herbal extracts, while the control group received petroleum gel. All participants applied the assigned product once daily before bedtime and avoided other lip products throughout the study.

### 2.7 Outcome Assessment

#### 2.7.1 Clinical Assessment

**FIGURE 2:**
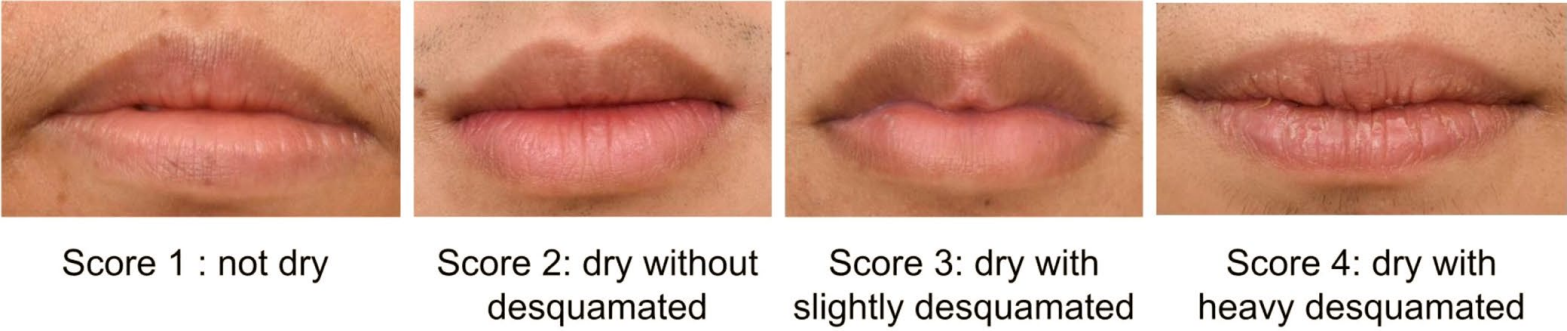
Lip roughness score.

**FIGURE 3:**
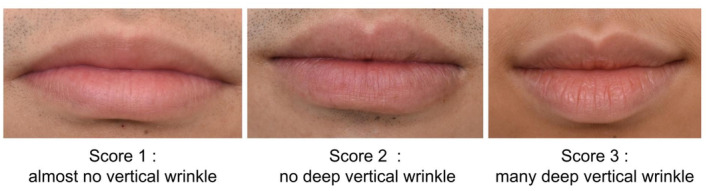
Lip wrinkle score.

**FIGURE 4:**
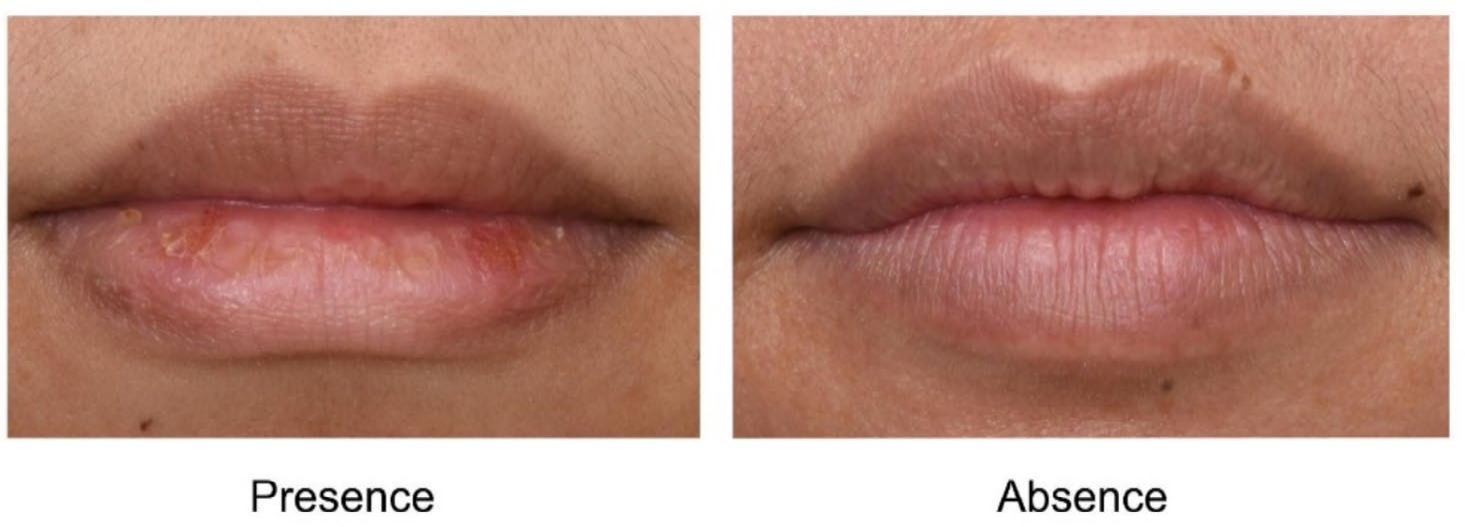
Chapped lip score.

Clinical assessments were conducted at baseline (day 0) and day 28 by well‐trained staff at the Dermatology Research Center Clinic, King Chulalongkorn Memorial Hospital. Participants acclimated for 15 min in an air‐conditioned room at 25°C before assessment. Lip data were collected using three devices: Antera 3D (Miravex, Ireland) to measure lip wrinkles, texture, melanin, and hemoglobin levels; professional photography; and Fotofinder (FotoFinder Systems GmbH, Germany) for microscopic lip imaging. Investigators (P.W., N.A., P.Sun., W.P.) evaluated lip roughness, wrinkles, and chapped lips using a modified visual assessment scale (modified from [18])); lip roughness was scored in four levels, severity of lip wrinkles was scored in three levels, as shown in Figures 2 and 3, respectively. Finally, chapped lip was scored in two levels as presence or absence (Figure 4).

#### 2.7.2 Participant Assessment

Participant assessments were evaluated via a self‐reported online questionnaire on day 0, 1, and 28 (Appendix 1). Participants rated lip dryness (0–10 numerical rating scale), satisfaction with lip appearance (score 1–4), overall satisfaction with the product (score 1–3), and specific product properties (score 1–5).

### 2.8 Statistical Analysis

In vitro assessment, the results were demonstrated as the mean ± SD of at least three independent determinations performed in triplicate. For two‐group comparisons, a one‐sample *t*‐test was calculated using the SPSS software program version 28 (SPSS Inc., Chicago, IL, USA). Statistical significance was considered at *p* < 0.05. GraphPad Prism 5 was used to create graphs for all experiments.

SPSS for Windows version 28.0 (IBM, USA) was used to analyze clinical data at a significance level of 0.05. Descriptive analyses of baseline demographic and lip characteristics were summarized using frequencies and percentages for categorical variables, while means and standard deviations (SD) were used for continuous variables. Changes in roughness level, wrinkle level, and presence of chapped lips based on visual assessment were analyzed within each group from baseline to day 28 using the Wilcoxon signed‐rank test. Comparisons between the control and test groups were performed using the Mann–Whitney *U* test.

Normality tests for wrinkle, roughness, melanin, and hemoglobin levels from the Antera 3DTM were conducted using the Kolmogorov–Smirnov test. Comparisons between baseline and day 28 within each group were performed using paired *t*‐tests for normally distributed data (including all control group data, as well as wrinkle and melanin levels in the test group). The Wilcoxon signed‐rank test was applied for the analysis of texture and hemoglobin levels in the test group. Clinical data comparisons between the control and lip hydrant groups at day 28 were assessed using an independent *t*‐test. Perceptive lip dryness scores were analyzed using the Friedman test for within‐group comparisons (baseline vs. day 28) and the Mann–Whitney *U* test for between‐group comparisons at baseline and day 28.

## 3 Results

### 3.1 Herbal Extracts Strengthen Skin Barrier by Antioxidation and Stemness Induction in Human Keratinocyte Cells

#### 3.1.1 Effect of Herbal Extracts on Viability of Human Keratinocyte Cells

**FIGURE 5:**
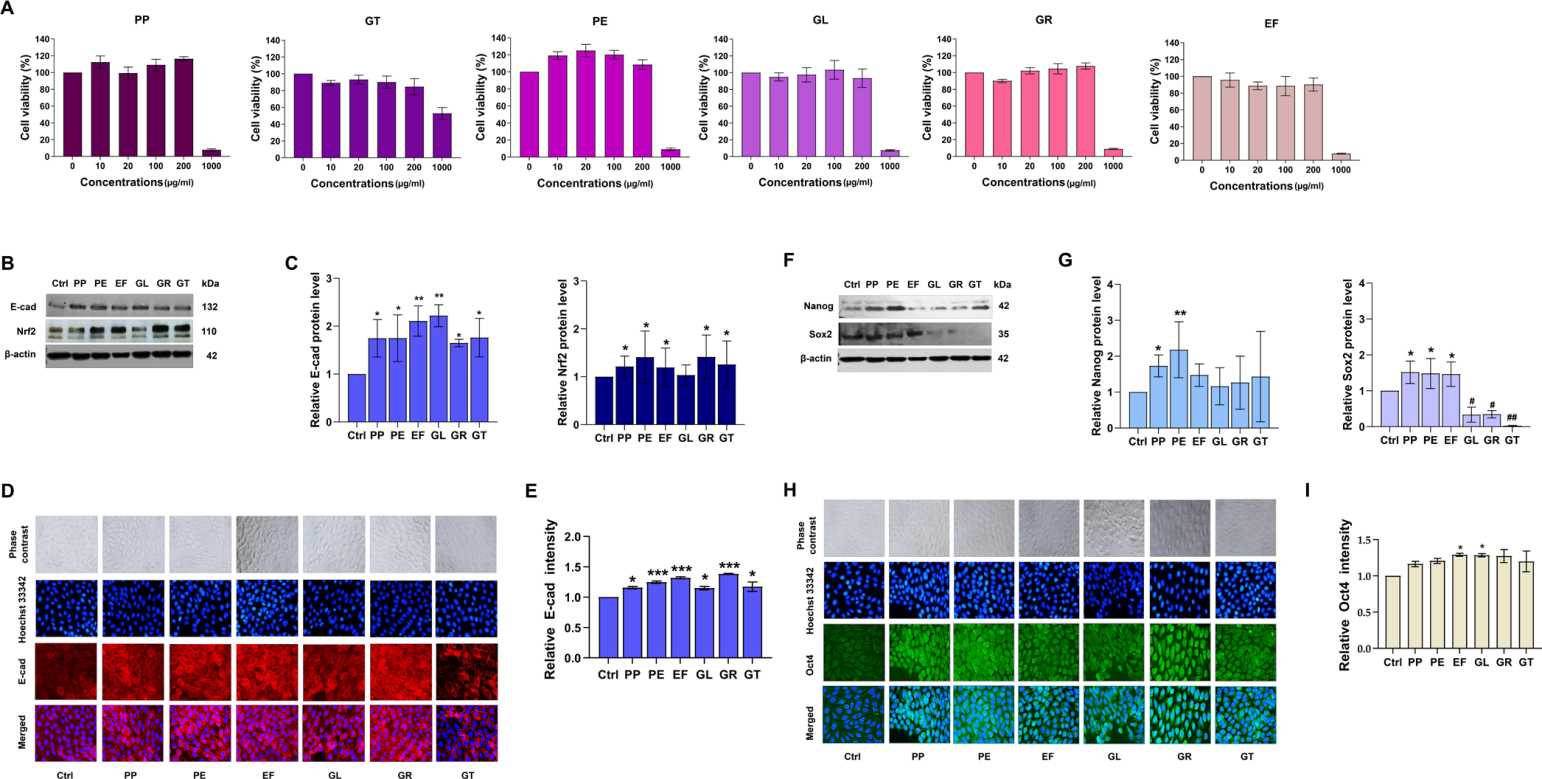
The effect of six herbal extracts on the viability of keratinocyte (HaCaT) cells determined by MTT assay. (A) Cell viability of HaCaT cells was determined after being treated with six herbal extracts, PP, PE, EF, GL, GR, and GT (0–1000 μg/mL) for 24 h. (B, C) Effect of the herbal extracts on expression and localization of cell adhesion E‐cadherin. HaCaT cells were treated with six herbal extracts (200 μg/mL) for 24 h. Then, they were subjected to determine the protein level by western blot. β‐Actin expression was used to confirm equal loading of protein. (D, E) The fluorescence intensity of E‐cadherin was also evaluated. (F, G) Effect of herbal extracts on Nrf2 expression. HaCaT cells were treated with six extracts (200 μg/mL) for 30 min. Then, the cells were subjected to determine the Nrf2 protein level using western blot analysis. β‐Actin expression was used to confirm equal loading of protein. (H, I) Effect of the herbal extracts on stem cell pluripotency factors. HaCaT cells were treated with six extracts (200 μg/mL) for 24 h. Then, the cells were subjected to determine Nanog and Sox2 expression using western blot analysis. β‐Actin expression was used to confirm equal loading of protein. The cells were treated with six herbal extracts for 24 h before stained immunocytochemistry for Oct4. The fluorescence intensity was calculated. The data was presented in mean ± SD. (*n* = 3) (*0.01 ≤ *p* < 0.05, **0.001 ≤ *p* < 0.01 and ***p < 0.001 compared with the non‐treated control).

To evaluate the effects of herbal extracts, the optimal conditions for six herbal extracts were tested in human keratinocyte cells. For cell viability assay, the cells were cultured and treated with the extracts, peppermint oil (PP), perilla extract (PE), emblica fruit extract (EF), galanga rhizome extract (GR), green tea leaf extract (GT), and guava leaf extract (GL) at concentrations ranging from 0 to 1000 μg/mL, and cell viability was assessed after 24 h of exposure to the extracts using the MTT assay. The results extremely showed a decrease in cell viability at 1000 μg/mL for all herbal extracts (Figure 5A). Therefore, the maximum concentration for further experiments was set at 200 μg/mL, to avoid direct toxicity of the extracts.

#### 3.1.2 Plant Extracts Increase Expression of Cell Adhesion E‐Cadherin

As E‐cadherin is essential for the proper epidermal barrier function [19], we first tested whether these plant‐derived extracts could benefit barrier function by enhancing the expression of cellular E‐cadherin in keratinocytes. The keratinocytes were incubated with non‐toxic concentrations (200 μg/mL) of six herbal extracts, and the expression of adhesive markers, E‐cadherin, was investigated by Western blot analysis. Figure 5B,C shows that all selected herbal extracts could be able to enhance the cellular level of E‐cadherin. E‐cadherin protein level was significantly increased in six herbal extract treatments when compared with non‐treated control (Figure 5C). To confirm the upregulation of E‐cadherin, immunocytochemistry with E‐cadherin‐specific antibody was performed. After 24 h‐treatment, E‐cadherin expression was notably upregulated in the extract‐treated cells. The localization of the E‐cadherin expression in all treated groups was intensified at the edge of the cells (Figure 5D,E), implying the enhancement of cell‐to‐cell junction. These data indicated that these herbal extracts increased the adhesion strength of keratinocyte cells.

### 3.2 Herbal Extracts Increase Cellular Antioxidant Machinery via Upregulation of Nuclear Factor Erythroid‐2‐Related Factor (Nrf2)

Moreover, one of the key cellular antioxidative stress responses is the nuclear factor erythroid 2‐related factor 2 (Nrf2) signaling pathway. Nrf2 is a crucial transcription factor that induces cytoprotective genes in response to reactive oxygen species (ROS). Inadequate ROS neutralization is linked to undesirable skin changes associated with aging and disease [20]. Cells were treated with non‐toxic concentrations (200 μg/mL) of herbal extracts for 30 min, as previously described. After treatment, Nrf2 levels were assessed by Western blotting. The results showed a significant increase in Nrf2 expression with PP, PE, EF, GR, and GT but no change with GL compared to the untreated control (Figure 5E,F). These findings suggest that the five Thai herbal extracts—PP, PE, EF, GR, and GT—exhibit antioxidant activity.

#### 3.2.1 The Extracts Showed Potential to Enhance Stem Cell Factors

Since the stemness of the epidermal layer is linked to maintaining epithelial barrier functions, we investigated the effect of the extracts on the stemness of human keratinocytes. Stem cell properties are determined by the levels of pluripotency factors such as Nanog, Oct4, and Sox2 [21]. We assessed these factors in keratinocyte cells treated with extracts at a concentration of 200 μg/mL. Results revealed a significant increase in Sox2 in the cells exposed to PP, PE, and EF while demonstrating a significant decrease when treated with GL, GR, and GT. Nanog was notably upregulated in response to the treatment with PP and PE, while Oct4 was significantly elevated in response to EF and GL (Figure 5H,I). This data indicates that PP, PE, and EF upregulate at least two stem cell self‐renewal markers together with upregulation of E‐cadherin and Nrf2. Consequently, we selected these three herbal extracts as a basal mixture for further formula development.

#### 3.2.2 Effect of Various Formula of Herbal Mixtures on Human Keratinocyte Cells

**FIGURE 6:**
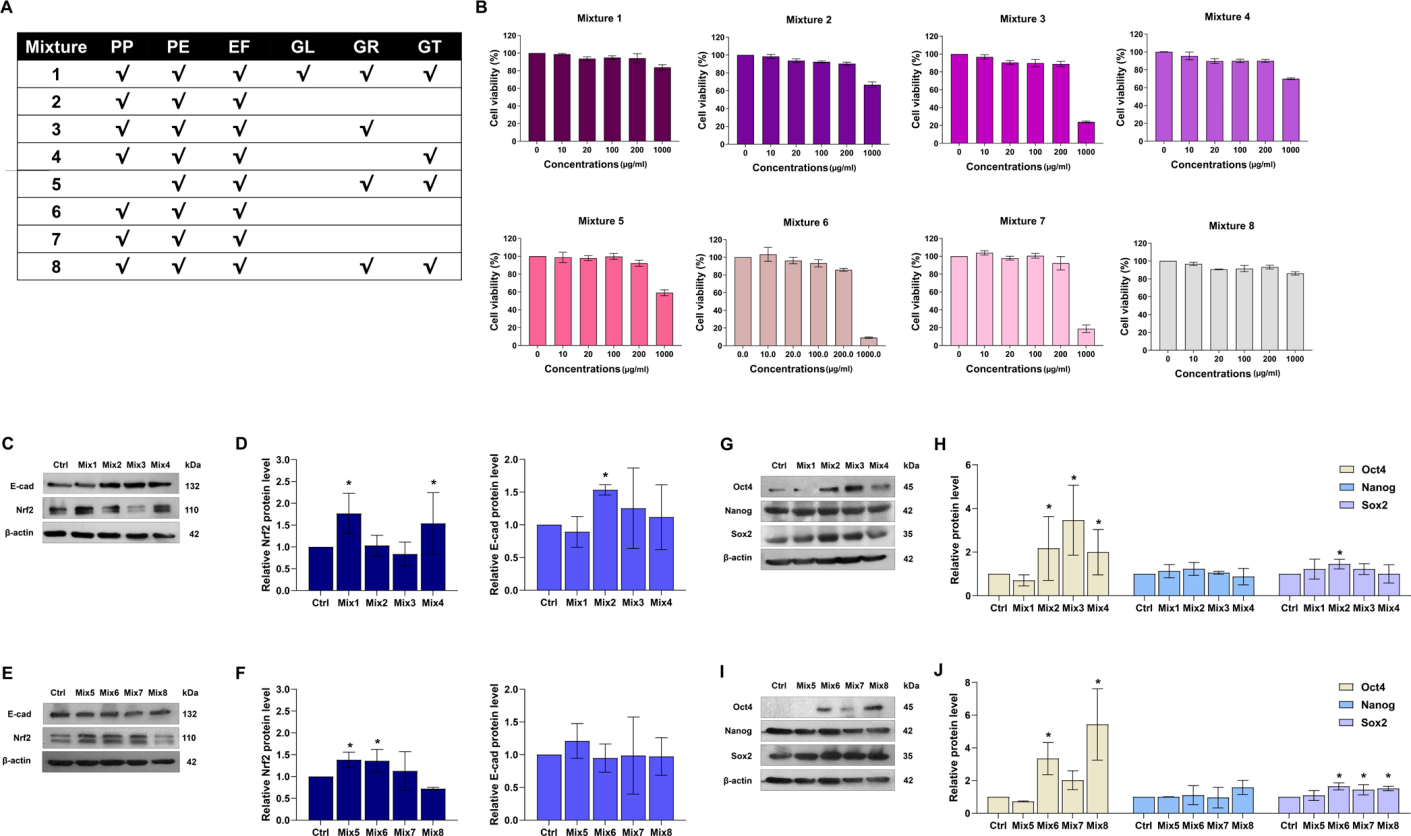
The six herbal extracts were mixed as eight mixtures which contained different extracts and proportions. (A) The table demonstrates the composition of each mixture. (B) The cytotoxic of each mixture was evaluated by MTT assay. The cell viability was calculated after treatment of various concentrations of mixtures for 24 h in HaCaT cells. (C, D) Effect of the herbal mixtures on expression of cell adhesion E‐cadherin. HaCaT cells were treated with eight mixtures (200 μg/mL) for 24 h. Then, they were subjected to determine the protein level by western blot. β‐Actin expression was used to confirm equal loading of protein. (C‐F) Effect of herbal extracts on Nrf2 expression. HaCaT cells were treated with eight mixtures (200 μg/mL) for 30 min. Then, the cells were subjected to determine the Nrf2 protein level using western blot analysis. β‐Actin expression was used to confirm equal loading of protein. (G‐ J) Effect of the herbal mixtures on stem cell pluripotency factors. HaCaT cells were treated with eight mixtures (200 μg/mL) for 24 h. Then, the cells were subjected to determine Oct4, Nanog, and Sox2 expression using western blot analysis. β‐Actin expression was used to confirm equal loading of protein. (*n* = 3) (*0.01 ≤ *p* < 0.05 compared with the non‐treated control).

After we determined the potential of each herbal extract, we decided to combine herbal extracts to be eight mixtures (Mix1‐Mix8). The composition of each herbal mixture is shown in Figure 6A. The cell viability of each herbal mixture was evaluated using MTT assay. The cells were treated with various concentrations of each mixture (0–1000 μg/mL) for 24 h. Same as herbal extracts, the cell viability was strongly decreased when treated with concentration of 1000 μg/mL (Figure 6B). Therefore, further experiments were conducted at concentration 200 μg/mL of herbal mixtures.

We evaluated the effect of various mixtures in the same manner as herbal extracts. All significant protein markers were examined using western blot analysis. E‐cadherin protein level was notably upregulated only after treated with Mix2 for 24 h when compared with non‐treated control (Figure 6C,D), while antioxidative marker Nrf2 significantly differed in Mix1‐, Mix4‐, Mix5‐, and Mix6‐treatment (Figure 6C–F). Moreover, we assessed pluripotency factors Nanog, Oct4, and Sox2 in keratinocyte cells treated with mixtures at a concentration of 200 μg/mL. Results revealed a significant increase in Oct4 in the cells exposed to Mix2, Mix3, Mix4, Mix6, and Mix6. Sox2 was notably elevated in response to the treatment of Mix2, Mix6, Mix7, and Mix8 while Nanog was not altered in all treatments (Figure 6G–J). Taking together, Mix2 demonstrated the most effective against HaCaT cells. Therefore, this mixture was selected for further formulation in clinical trials.

### 3.3 Clinical Evaluation

#### 3.3.1 Participant Characteristics

A total of 66 participants, comprising 42 females and 24 males with an average age of 28.52 ± 6.33 years, completed the study. The most common lip complaints reported were dry lips (*N* = 61), chapped lips (*N* = 43), dark lips (*N* = 26), and lip wrinkles (*N* = 20). Baseline demographic and lip characteristics of the participants are summarized in Table 2.

**TABLE 2:** Baseline demographic and lip characteristics of study participants.

Characteristics	Total (*N* = 66)	Test group (*n* = 33)	Control group (*n* = 33)	*p*
Age (mean ± SD)	28.52 ± 6.33	27.36 ± 5.94	29.67 ± 6.68	0.13
Female: *N* (%)	42 (63.64)	21 (63.64)	21 (63.64)	—
Numerical rating scale‐lip dryness (mean ± SD)	6.09 ± 1.98	6.09 ± 1.99	6.09 ± 2.04	0.79
Lip roughness score: *N* (%)				0.03
1 (Not dry)	1 (1.52)	1 (3.03)	0 (0)	
2 (Dry without desquamation)	18 (27.27)	14 (42.43)	4 (12.12)	
3 (Dry with slight desquamation)	37 (56.06)	13 (39.39)	24 (72.73)	
4 (Dry with heavy desquamation)	10 (15.15)	5 (15.15)	5 (15.15)	
Lip wrinkle score: *N* (%)				0.54
1 (Almost no vertical wrinkles)	4 (6.06)	0	4 (12.12)	
2 (No deep vertical wrinkles)	31 (46.97)	20 (60.61)	11 (33.33)	
3 (Deep vertical wrinkles)	31 (46.97)	13 (39.39)	18 (54.55)	
Presence of chapped lips: *N* (%)	27 (40.9)	13 (39.39)	14 (42.42)	0.80

#### 3.3.2 Visual Assessment

**FIGURE 7:**
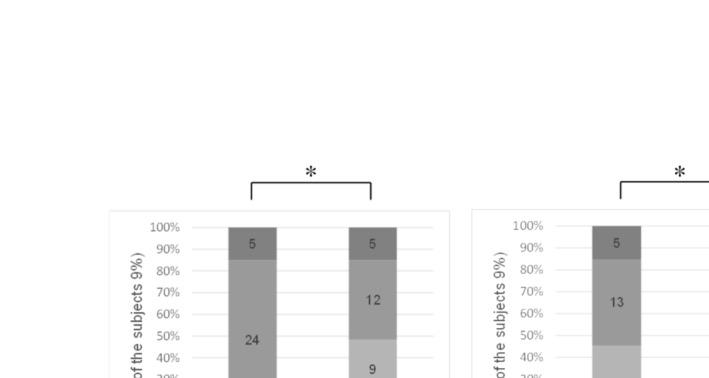
Changes in levels of lip roughness from visual assessment scale (*p < 0.05, Wilcoxon signed‐rank test).

**FIGURE 8:**
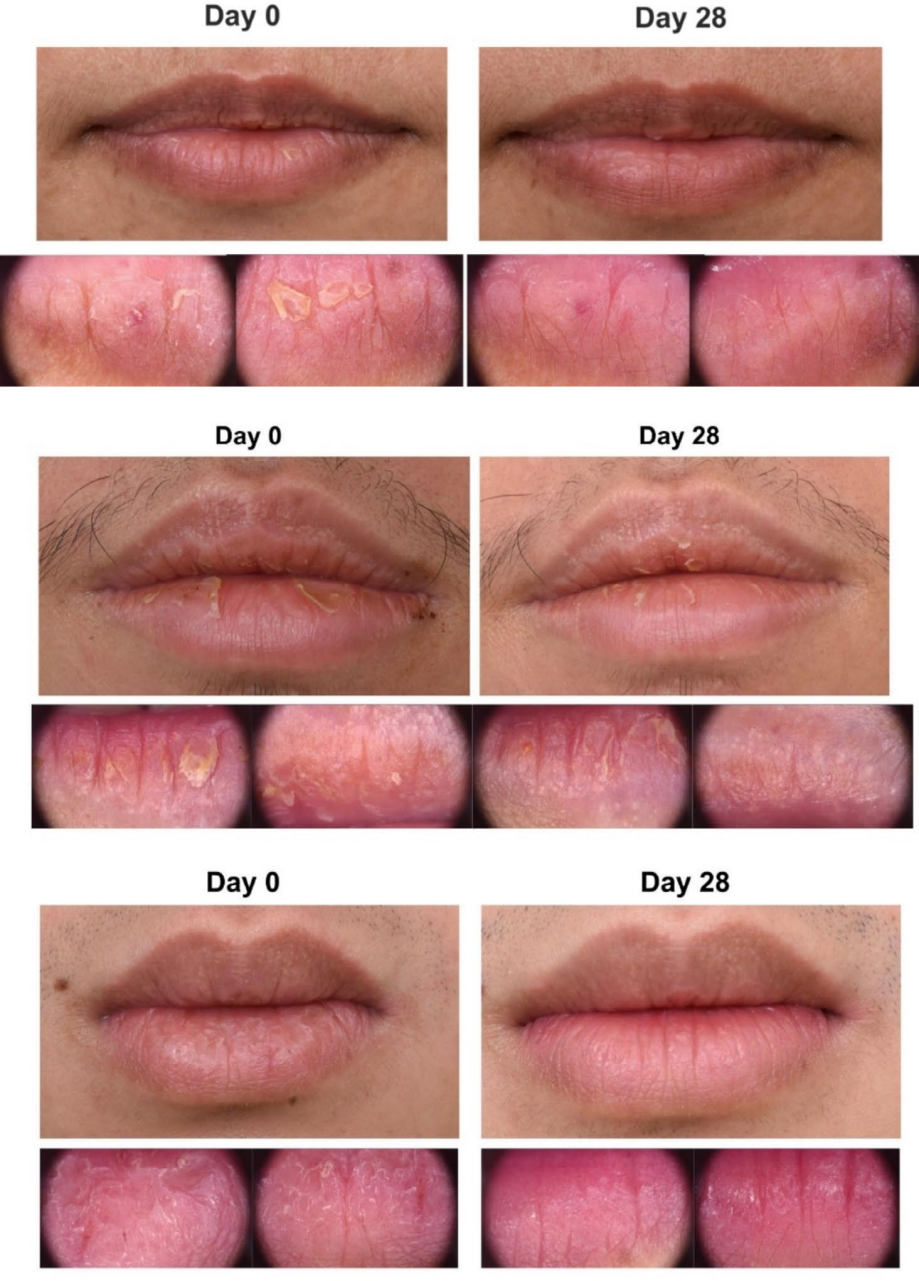
Significant reduction in lip desquamation and scaling after 28‐day application of lip hydrant in the test group.

Visual assessments showed a statistically significant reduction in lip roughness from baseline to day 28 within both the test and control groups (*p* = 0.032 and *p* = 0.04, respectively). However, no significant differences were observed between the two groups (Figure 7). While the severity of lip wrinkles did not change significantly within either group, the chapped lip score was significantly lower in the test group compared to the control group (*p* < 0.05). Photographs and microscopic images from the Fotofinder system revealed noticeable improvements in lip appearance for participants in the test group by day 28 (Figure 8).

#### 3.3.3 Objective Lip Analysis

Antera 3D analysis did not show significant differences in wrinkle, texture, melanin, or hemoglobin levels within or between the groups (Table 3).

**TABLE 3:** Mean wrinkle, texture, melanin, and hemoglobin level from Antera 3D.

Measurement, mean	Control group		Test group	
	Baseline	Day 28	Baseline	Day 28
Wrinkle	14.63	14.59	13.99	13.87
Texture	13.98	13.92	13.19	13.65
Melanin	0.73	0.73	0.74	0.74
Hemoglobin	2.14	2.18	2.12	2.13

#### 3.3.4 Participant Assessment

**FIGURE 9:**
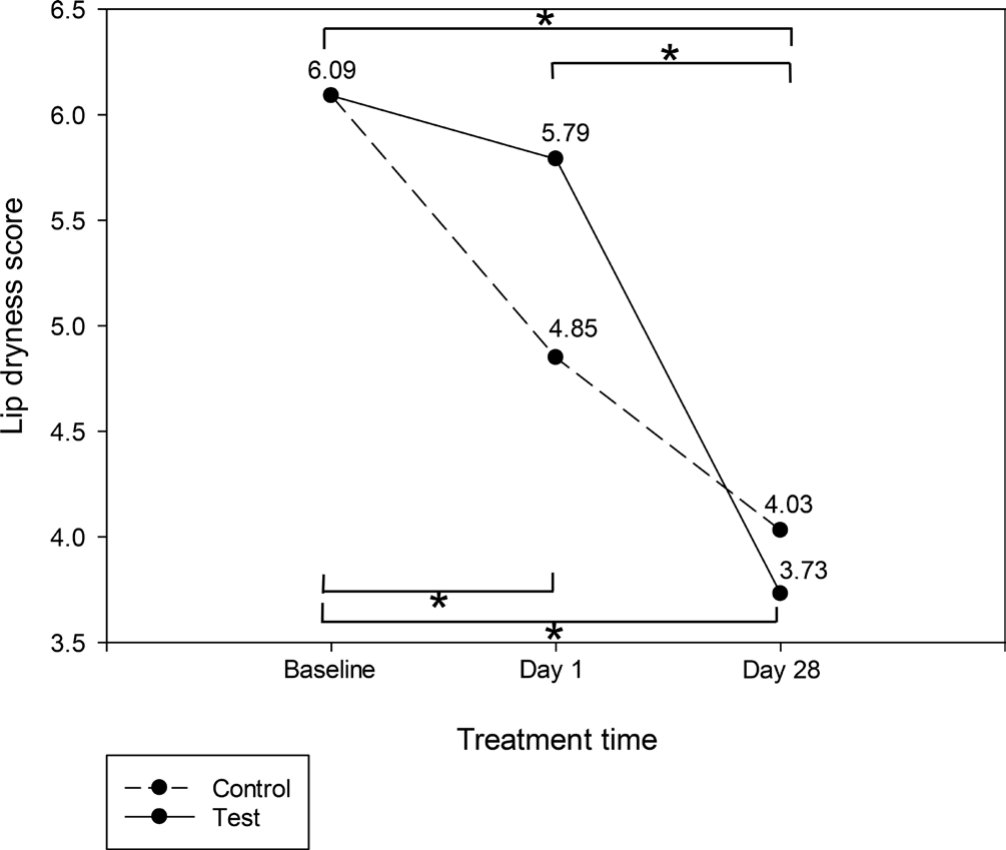
Reduction in perceived lip dryness score in test and control group (*p < 0.05, Friedman's test).

**FIGURE 10:**
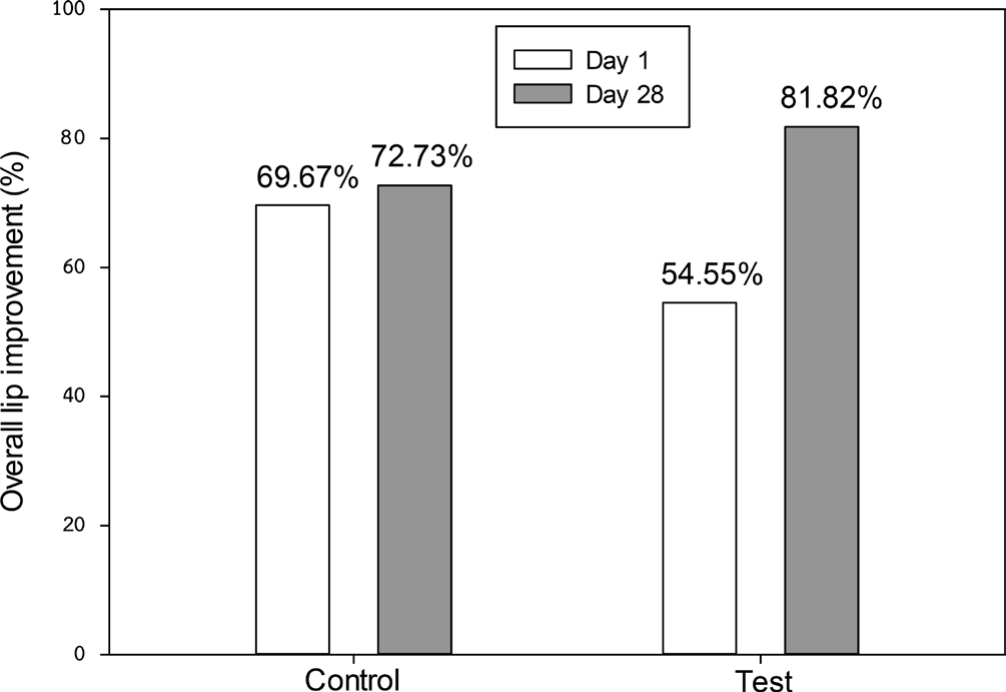
Subjective self‐assessment of overall lip improvement (% subjects marked as slightly change and significant change).

Self‐assessments indicated that participants in the test group reported a significant improvement in perceived lip dryness from baseline to day 28 (*p* < 0.01), though no significant differences were noted between the test and control groups on days 1 and 28 (Figure 9). Additionally, the percentage of participants in the test group who reported overall lip improvement increased significantly from 54.55% on day 1 to 81.82% on day 28 (*p* < 0.05), compared to a smaller increase in the control group (69.67%–72.73%) (Figure 10).

#### 3.3.5 Product Satisfaction

**FIGURE 11:**
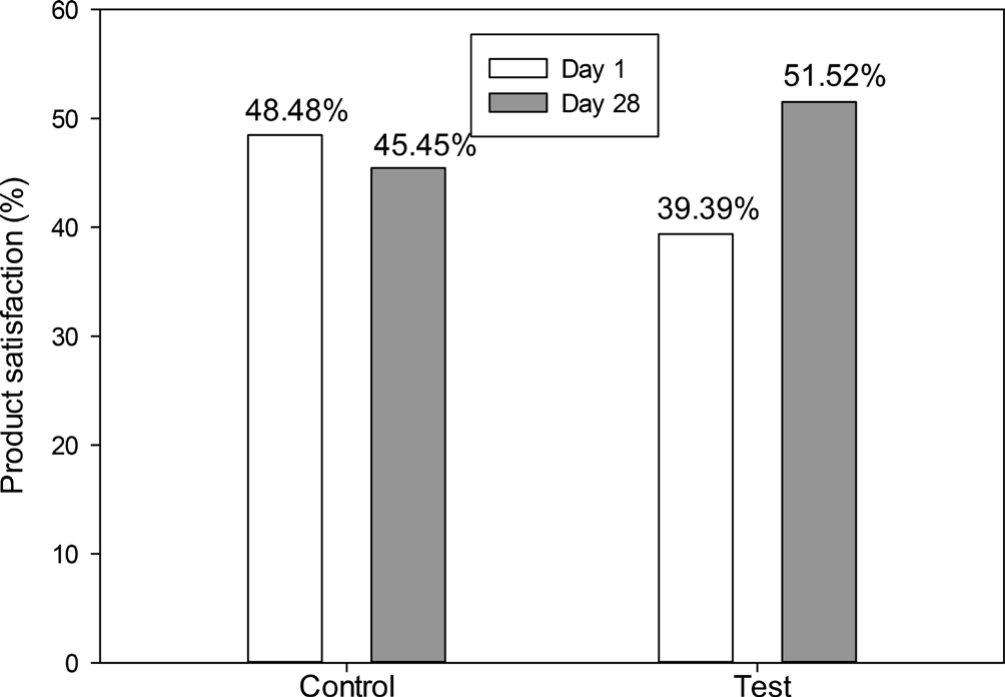
Percentage of overall satisfaction with the lip product.

While overall satisfaction with the lip product increased in the test group from 39.39% on day 1 to 51.52% on day 28, satisfaction in the control group decreased slightly from 48.48% to 45.45% (Figure 11). Reports of irritation and swelling were minimal, with two participants in the test group experiencing symptoms on day 1 and one participant on day 28, compared to one participant in the control group on day 28. Both groups showed equal interest in purchasing the product, with 45.45% indicating they would buy it if available.

## 4 Discussion

This study aimed to evaluate the therapeutic potential of a novel polyherbal lip hydrant in improving lip hydration and addressing common lip concerns such as dryness, roughness, and chapping. The findings suggest that the polyherbal formulation offers significant benefits in reducing lip roughness and alleviating chapped lips, particularly in comparison to the control group using petroleum gel.

Firstly, we selected the six herbal extracts which are commonly used in cosmeceuticals due to their antioxidant and anti‐inflammatory activity [22, 23, 24, 25, 26]. The concentration at 200 μg/mL was used in activity evaluations due to the cytotoxic potency (Figure 5A). The epidermis is a stratified squamous with multiple layers. It acts as the first barrier against the outside environment. E‐cadherin is found in all epidermis layers and plays a crucial role in tight junctional architecture. Upregulation of E‐cadherin might strengthen skin barriers [19]. In Figure 5B–E, E‐cadherin protein level was significantly upregulated after extract treatment for 24 h. Interestingly, most of the E‐cadherin in the treatment group was intensely located on the cell surface membrane (Figure 5D). This was supported that the extracts could strengthen the tight junction of the keratinocyte cells. The stronger skin barrier not only protects the harmful from environment but also prevents water loss which is the cause of lip dryness. Moreover, the essential factor that influences skin damage is oxidative stress which is caused by reactive oxygen species (ROS). Nrf2 signaling pathway is one of the main mechanisms to fight against excessive exposure to ROS overwhelms the antioxidative mechanism, ROS will destroy the essential macromolecules such as DNA, signaling protein for survival, and genetics which leads to skin damage and skin cancer [27, 28]. Therefore, reinforcement of the antioxidative mechanism like Nrf2 will be the promising strategy. The results demonstrated that the Nrf2 protein level was significantly elevated after herbal extract treatment except in GL treatment group (Figure 5F,G). At this point, selected herbal extracts have the potential of strengthening and protecting the skin. Figure 5H–K, Oct4 was notably upregulated in EF and GL while Nanog was remarkably elevated in PP and PE treatment. Sox2 was significantly increased in all extract treatments. Since in the skin, there are small populations of stem cells located in the thin layer of epidermis. This population is responsible for regeneration of the epidermis cells. Oct4, Nanog, and Sox2 are the main transcription factors in this self‐renewal mechanism [9, 29]. PP, PE, and EF are the most effective extracts in previous experiments. Therefore, all the mixtures contain these three extracts but different proportions of each extract. In Figure 6, the results revealed the potential of the mixture against tight junction strengthening, antioxidant activity, and self‐renewal capacity. High expression of E‐cadherin is known to strengthen tight junctions between epithelial cells, enhancing the skin barrier [10]. Additionally, self‐renewal activity is linked to the self‐healing ability of the lip epithelium [30]. In cases of lip cheilitis, most patients experience severely dry and cracked lips. Therefore, the primary issue to address is strengthening and repairing the damaged lips. Although Mix2 lacks antioxidant activity, it is the only formulation with potential for upregulating E‐cadherin and enhancing self‐renewal capacity, as shown in Figure 6. Thus, Mix2 was chosen for the lip formulation in the clinical trial.

The observed improvements in lip condition, especially in the test group, can be attributed to the antioxidant and anti‐inflammatory properties of the herbal extracts used in the lip hydrant. For instance, Perilla extract contains polyphenols, rosmarinic acid, and caffeic acid, which have been shown to enhance skin hydration by upregulating hyaluronan synthase 2 (HAS2) and hyaluronan synthase 3 (HAS3), enzymes responsible for hyaluronic acid production [31]. This mechanism likely contributed to the reduction in lip roughness observed in the study. These results align with existing literature, which supports the use of hyaluronic acid and related compounds in enhancing skin hydration and elasticity [32, 33].

Emblica fruit extract, known for its high antioxidant content, has demonstrated anti‐aging effects by promoting type‐1 pro‐collagen levels and inhibiting collagenase and matrix metalloproteinase (MMP) activity [34]. Additionally, it has anti‐inflammatory properties, evidenced by the downregulation of genes such as COX‐2, iNOS, IL‐16, and IL‐6 [35]. These effects likely contributed to the observed improvements in lip smoothness and overall lip condition among participants in the test group.

The results of this study are consistent with previous research that has highlighted the benefits of herbal extracts in skincare and lip care. While earlier studies have primarily focused on visual assessments of lip condition [32, 33, 36, 37, 38], this study also utilized advanced imaging techniques such as Antera 3D and Fotofinder to provide a more comprehensive evaluation. While traditionally employed in clinical research for studying skin diseases, skin cancer, and hair analysis [39, 40, 41], this is, to our knowledge, the first instance of Fotofinder application in lip analysis. This approach allowed for the capture of high‐resolution images and provided detailed analyses of the lip surface. The application of Antera 3D technology for measuring enhancements in lip condition has only been reported in one prior investigation [38]. Despite the lack of statistically significant differences in objective measures such as wrinkle depth and texture, the observed trends towards improvement suggest a potential benefit of the polyherbal lip hydrant. However, these findings warrant further investigation through longer‐term studies to conclusively determine the effects of prolonged use.

The positive feedback from participants further underscores the potential of the polyherbal formulation as an effective lip care product. The increase in participant‐reported satisfaction and lip improvement over the course of the study indicates that the product is well‐received and may offer a viable alternative to conventional lip care products, particularly for individuals seeking natural and effective solutions.

While this study presents compelling evidence for the efficacy of the polyherbal lip hydrant in improving lip hydration, several limitations should be noted. The study did not employ Trans‐epidermal Water Loss (TEWL) measurements due to the inherent challenges associated with this method in lip assessments. However, future studies should consider incorporating alternative methodologies that can accurately capture lip hydration levels to further validate and strengthen the findings. The participant's age was restricted to 40 years to mitigate potential confounding factors associated with aging and comorbidities. Additionally, the study duration was limited to 4 weeks to ensure participant engagement and minimize dropout rates. To enhance the generalizability of these findings, future research should consider expanding the inclusion criteria to encompass a broader participant population, including older individuals and greater diversity. Furthermore, extending the study duration would provide valuable insights into the long‐term benefits and optimal usage patterns of the product.

## 5 Conclusion

The study findings provided compelling evidence supporting the therapeutic potential of the novel polyherbal lip hydrant in enhancing lip hydration, reducing roughness, alleviating chapped lip, and also improving overall lip condition. The product was well‐received by Thai adults. Further research is needed to explore the long‐term effects of the product.

## Author Contributions

**Pimchanok Sutthiboonyapan:** contributed to conceptualization, methodology, funding acquisition, formal analysis, visualization, and writing the original draft of the article. **Nicharat Sriratanasak:** data analysis, data curation, visualization, and writing the original draft of the article. **Bhurichaya Innets:** investigation, data analysis, data curation, visualization, and writing the original draft of the article. **Nachon Angkanaporn:** contributed to the data curation, investigation, and final approval of the manuscript. **Parinnapa Suntornchot:** contributed to the data curation, investigation, and final approval of the manuscript. **Warunya Panyain:** contributed to the data curation, investigation, and final approval of the manuscript. **Thantrira Porntaveetus:** contributed to conceptualization, and critical revision of the manuscript. **Paswach Wiriyakijja:** contributed to the conceptualization, methodology, formal analysis, writing the original draft of the article, and critical revision of the manuscript. **Pithi Chanvorachote:** contributed to the conceptualization, methodology, formal analysis, writing the original draft of the article, and critical revision of the manuscript.

## Ethics Statement

This study was approved by the Human Research Ethics Committee of the Faculty of Dentistry, Chulalongkorn University, Thailand (HREC‐DCU 2023‐060).

## Conflicts of Interest

The authors declare no conflicts of interest.

## Data Availability

The authors confirm that the data supporting the findings of this study are available within the article.

## Appendix 1 A Self‐Reported Questionnaire for Participant Assessment

Please answer the following questions based on your opinion.

1. How dry is your lip? (numerical number 0–10; 0 = not dry to 10 = very dry)
2. How do you feel about your lip condition? (1 = aggravated, 2 = unchanged, 3 = slightly improved, 4 = markedly improved)
3. Do you like the product? (1 = no, 2 = neutral, 3 = yes)

Please rate the following statements. (1 = strongly disagree, 2 = disagree, 3 = neutral, 4 = agree, 5 = strongly agree).

### Product‐Specific Properties

1. The product moisturizes your lip and prevents lip dryness.
2. The product enhances your lip color.
3. The product helps reduce fine lines.
4. The product helps your lip look and feel healthier.
5. The product is not oily or greasy.
6. The product is suitable for daily use.
7. You will use the product if it is available.
